# Polypharmacy among Underserved Older African American Adults

**DOI:** 10.1155/2017/6026358

**Published:** 2017-05-23

**Authors:** Mohsen Bazargan, James Smith, Masoud Movassaghi, David Martins, Hamed Yazdanshenas, Seyede Salehe Mortazavi, Gail Orum

**Affiliations:** ^1^Charles R. Drew University of Medicine and Science (CDU), Los Angeles, CA, USA; ^2^University of California, Los Angeles (UCLA), Los Angeles, CA, USA; ^3^Mental Health Research Center, Tehran Institute of Psychiatry, School of Behavioral Sciences and Mental Health, Iran University of Medical Sciences, Tehran, Iran; ^4^Keck Graduate Institute, School of Pharmacy, Claremont, CA, USA

## Abstract

The purpose of the present study was to examine correlates of polypharmacy among underserved community-dwelling older African American adults.* Methods*. This study recruited 400 underserved older African Americans adults living in South Los Angeles. The structured face-to-face interviews collected data on participants' characteristics and elicited data pertaining to the type, frequency, dosage, and indications of all medications used by participants.* Results*. Seventy-five and thirty percent of participants take at least five and ten medications per day, respectively. Thirty-eight percent of participants received prescription medications from at least three providers. Inappropriate drug use occurred among seventy percent of the participants. Multivariate analysis showed that number of providers was the strongest correlate of polypharmacy. Moreover, data show that gender, comorbidity, and potentially inappropriate medication use are other major correlates of polypharmacy.* Conclusions*. This study shows a high rate of polypharmacy and potentially inappropriate medication use among underserved older African American adults. We documented strong associations between polypharmacy and use of potentially inappropriate medications, comorbidities, and having multiple providers. Polypharmacy and potentially inappropriate medications may be attributed to poor coordination and management of medications among providers and pharmacists. There is an urgent need to develop innovative and effective strategies to reduce inappropriate polypharmacy and potentially inappropriate medication in underserved elderly minority populations.

## 1. Introduction

Almost 72% of elderly Medicare beneficiaries have at least two medical chronic conditions, and 39% suffer from four or more chronic conditions [1]. Every year, Medicare beneficiaries with five or more chronic conditions see an average of thirteen physicians each and fill over fifty prescriptions [2]. Proper treatment of many medical conditions requires the use of medications that can be misused, abused, and/or lead to dependency. A recent study using the National Health and Nutrition Examination Survey (1988–2010) shows that, between 1988 and 2010, the median number of prescription medications (Rx) used among adults aged 65 and older doubled from 2 to 4, and the proportion taking at least 5 medications tripled from 12.8% to 39% [3].

Older persons with multiple chronic conditions often receive duplicate testing, conflicting treatment advice, and prescriptions that are contraindicated [4, 5]. Polypharmacy is a major problem that contributes to costs, adverse drug events, confusion, compliance issues, management errors, hospitalization, and premature mortality among the elderly [6–16]. Research has shown that those taking numerous medications have a higher likelihood of emergency room visits and hospitalizations [17, 18]. A recent study documented that older people who took multiple medications faced an increased risk of frailty and death, and for older individuals who took a higher number of medicines, each additional medicine incurred a 22% increase in the risk of transitioning from a state of robust health to dying over the three-year study period [19]. Despite awareness of polypharmacy and its potential consequences in older patients, polypharmacy remains widespread [20]. While Rx use increased dramatically within the last decade, studies also indicate that polypharmacy in the United States continues to increase and is affecting 33% to 60% of patients, and that multiple medication use is associated with a host of undesirable outcomes, including adverse drug reactions, geriatric syndromes, and poor adherence [7].

African Americans, compared to whites, are disproportionally affected by chronic medical conditions for which multiple prescriptions and treatments are required [21]. Almost 47% of elderly African American Medicare beneficiaries suffer from at least four chronic conditions, compared to 38% of their non-Hispanic white counterparts [1]. While underserved elderly African Americans have high-risk profiles for increased morbidity and disability, generally they receive less aggressive treatment and are less likely to be prescribed newer, more effective and simplified medication combinations [22–26]. Therefore, lack of access to combined and simpler medication regimens among underserved older African American adults likely results in the use of a higher number of older, generic medications with more complex dosing regimens. Indeed, systematic reviews of studies confirmed that simpler, less frequent dosing regimens resulted in better medication adherence across a variety of therapeutic classes [27].

Lack of equitable access to Rx among elderly African Americans, compared to their white counterparts, was noted two decades ago [28] yet remains a major concern [29]. Recent National data show that older African Americans are more likely to report not having a* “regular care provider”* or a* “regular place of care”* (noncoordinated care) than their white counterparts [30], which is associated with inappropriate use of Rx prescribed by multiple providers. Despite the introduction of a Medicare outpatient prescription drug benefit in January 2006, the longstanding Rx access gaps between elderly African Americans and whites remain unchanged, with three times as many elderly African American beneficiaries (18%) going without prescribed medication compared to white beneficiaries (6%) [31].

In summary, national studies show that race/ethnicity is associated with polypharmacy variation in middle age and older adults [32]. Indeed, polypharmacy, a common and important problem related to drug use, occurs subsequent to this multimorbidity in the elderly in all populations [33], however; there is a significant need for additional research to reduce the burden of medication mismanagement and polypharmacy among underserved older African American adults.

The goal of this study is to describe characteristics of underserved older African American adults engaging in polypharmacy. We defined polypharmacy merely based on the number of medications used by participants. Polypharmacy is characterized as the use of multiple medications for the treatment of a single, or several, coexisting diseases [33]. Additionally, correlates of medication use, including demographic characteristics, smoking status, alcohol use, number of providers (physicians and pharmacies), severity of pain, and number of potentially inappropriate medications (PIM) for older adults, are examined.

## 2. Methods

Details of the study design have been previously described [34]. In brief, data were collected between 2013 and 2014 from 400 community-dwelling, underserved, older adult, self-identified African Americans from 16 predominantly African American churches located in Service Planning Area 6 (SPA 6) of Los Angeles (LA) County. LA County has the largest population of any county in the nation. More than 1.3 million of the 10.3 million residents who live in LA County are 65 years and older [35]. A Service Planning Area, or SPA, is a specific geographic region within LA County. Due to the large size of LA County (4,300 square miles), it has been divided into 8 geographic areas [36]. These distinct regions allow the Department of Public Health to develop and provide more relevant public health and clinical services targeted to the specific health needs of the residents in these different areas. We specifically selected SPA 6 because the vast majority of elderly living in SPA 6 are African American (49%).

In addition to posting flyers announcing the proposed project at respective churches, the coordinator of this project assisted church leaders to convene pre- or post-Sunday sermon meetings to introduce the program project to the parishioners. Participants were encouraged to consider the potential benefits of this project to society. They were told that the information they provide may contribute to improving minority senior population health outcomes. In addition, they received a $20 remuneration fee. Two coauthors of this study, both trained physicians, conducted the face-to-face interviews in a private room at participating sites. Less than 5% (*n* = 19) of individuals who were approached refused to participate. Written informed consent was obtained from participants. This study was approved by the Charles R. Drew University of Medicine and Science Institutional Review Board.

### 2.1. Measurement

#### 2.1.1. Medication Use

Medication use was assessed by the drug inventory method. Participants were asked to bring all over-the-counter and prescription medications that they had taken in the two weeks prior to the interview. The interviewer transcribed from the container label the name of the medication, strength of the drug, expiration date, instructions, special warnings, providers' information, and so on.

#### 2.1.2. Potentially Inappropriate Medication (PIM) on Beers' List

The Beers list identifies medications and types of medications that are “potentially inappropriate” for older people. Using the 2012 American Geriatrics Society revised Beers Criteria [37], we documented the number of PIM use for each participant.

#### 2.1.3. Severity of Pain

Pain was measured using the revised version of Short-Form McGill Pain Questionnaire-2 (SF-MPQ-2) [38, 39]. Participants completed the SF-MPQ-2 by rating the extent to which they experienced each of 22 pain descriptors in the past week using an 11-point numeric rating scale (0 = “none” to 10 = “worst possible”). Severe pain was defined according to the World Health Organization scale, which was 7 to 10 on the 11-point numeric rating scale [40].

#### 2.1.4. Comorbidity

Participants self-reported “Yes” or “No” to a list of medical conditions, such as arthritis, back pain, kidney disease, stroke, and hypertension.

#### 2.1.5. Number of Providers and Copayment for Rx

Participants were asked if any copayment is required to fill a prescription. Number and description of pharmacies and providers used by participants were recorded from the label of medication containers.

#### 2.1.6. Smoking Status and Alcohol Use

Smoking status and alcohol use were computed from self-reporting during the past year. Smoking status was categorized into three groups: current, former, and never smoked. In addition, alcohol use was categorized based on number of drinks per day (none, 1-2, and ≥ 3 drinks per day).

#### 2.1.7. Statistical Analysis

Descriptive statistics were used to measure the frequency, mean, and standard deviation of all variables. Furthermore, we used the chi-square test to examine associations between medication used and demographic characteristics, comorbidities, and medication complications. In addition, multiple logistic regression was employed to examine the correlates of using medications (use of ≥ 5 medications versus fewer), adjusting for demographic characteristics and other relevant characteristics. We utilized a* p* value < 0.05 to identify statistically significant differences. To avoid multicollinearity, a diagnostic test was performed in multivariate analysis to examine intercorrelation among independent variables.

## 3. Results

### 3.1. Characteristics of Sample

This sample included 400 African American individuals who were between the ages of 65 to 94 years (Mean 73.5 ± 7). More than 21% were 80 years of age or older. One out of four participants reported having no high school diploma. Almost 65% of participants were women. Only 20% of the sample were currently married or lived with a companion.  Table 1 reports the percentage of self-reported chronic conditions among our sample. In all, the number of reported chronic illnesses ranged from zero to seventeen, with the average at just over five (5.2 ± 3.01). Nineteen percent of participants reported at least eight chronic conditions. Over 85% reported that they have been diagnosed with hypertension. One out of four individuals who were diagnosed with hypertension was using at least three antihypertensive agents from different classes. Almost 37% of participants were diagnosed with diabetes mellitus. Three out of four reported visiting more than one type of physician. Only 8% of respondents did not have a regular or primary care provider. Participants reported having an average of 6.71 scheduled physician visits within the past twelve months (range: 0 to 24; SD: 5.4). Thirty-eight percent of participants received Rx from at least three providers. Furthermore, the medication containers showed that 28% of participants used at least two pharmacies to fill their prescriptions.

Participants were taking an average of 5.7 (range: 0 to 18; SD: 3.02) prescription drugs. Twenty-three percent were using at least eight medications, whereas 37% and 40% of participants were taking five to seven and zero to four prescription medications, respectively. In addition, participants were taking an average of 1.25 over-the-counter medications. Using the 2012 American Geriatrics Society revised Beers Criteria [37], our data show that 27% and 43% of participants used at least one medication that was classified as “Avoid” and “Use Conditionally,” respectively. The most common drugs in the “Avoid” group were nifedipine 6% (24), diphenhydramine 3% (13), glyburide 2% (8), lorazepam 2% (8), cyclobenzaprine 2% (7), diazepam 2% (7), and digoxin hydroxyzine 2% (7).

### 3.2. Bivariate Correlates of Polypharmacy

Table 1 reports bivariate correlates of medication use with all other variables. The number of medications used was divided into two categories: “0–4” and “≥ 5” medications. Eight out of twelve variables were significantly associated with the number of medications used. This table reports that the proportions of older adults on 0–4 and ≥ 5 medications were 25% and 75%, respectively. Compared with those taking 0–4 medications, persons taking ≥ 5 medications were more likely to be women, 70–79 years of age, former smokers, visiting two or more providers, filling their prescriptions in different pharmacies, diagnosed with a higher number of comorbidities, suffering from a higher level of pain, and used a higher number of PIMs.

### 3.3. Multivariate Correlates of Medication Use

Table 2 reports the result of multiple logistic regression, adjusting for demographic characteristics including age, gender, education, and marital status. This table reports adjusted odd ratios (OR) between independent variables and two categories of medications used (“0–4” and “≥ 5” medications). Four variables were significantly associated with the use of medications. Adjusting for demographic characteristics and other related variables, number of providers was the strongest correlate of excessive use of medications. The odds of being in the group of survey respondents who used ≥5 medications were almost seven times (OR = 6.67; CI: 2.82–15.71) higher among individuals who received medical care from multiple providers. Participants with one or at least two PIMs were 2.24 (CI: 1.17–4.30) and 4.6 (CI: 2.10–10.04) times more likely to use ≥ 5 medications than their counterparts with no PIMs, respectively. Moreover, when demographic characteristics and other variables were adjusted, participants who were diagnosed with 4–7 or ≥8 comorbidities were 3.2 (CI: 1.72–5.94) and 6.11 (CI: 2.02–18.50) times more likely than their counterparts with less than ≤ 3 comorbidities to use ≥ 5 medications, respectively. Finally, gender was the only demographic variable that was associated with polypharmacy. The odds of being in the group of survey participants who used ≥ 5 medications were 2.36 (CI: 2.36–4.42) times higher among women than their male counterparts.

## 4. Discussion

We documented that polypharmacy remains an important issue among underserved older African American adults. The National Health and Nutrition Examination Survey (NHANES) shows 39% (CI: 35.8, 42.3) of older adults aged 65 years and older are taking ≥ 5 medications [3], whereas our data show 75% of participants currently taking ≥ 5 medications and 30% taking ≥ 10 medications. The high rate of polypharmacy documented in our study may reflect the compromised level of care for the senior population residing in SPA 6 of LA County. Home to over one million residents, SPA 6 is disproportionately harmed by health disparities compared to the rest of LA County [41].

A recent systematic review and meta-analysis of 25 studies targeting older adults with polypharmacy (≥ 4 drugs) found no convincing evidence that the strategies assessed in these studies were effective in reducing polypharmacy or had an impact on clinically relevant endpoints [42]. Even though interventional strategies employed in these studies were complex, it is unclear yet how to best organize and implement them to achieve a reduction of inappropriate polypharmacy [42]. Additional investigational and interventional studies are needed to offer practical solutions to reduce polypharmacy, PIM use, and other medication related complications.

Our study echoes previous research that shows that one of the important correlates of inappropriate prescription medication use in older patients is the number of prescribed medications [43–46]. Our data show that 70% of the sample used at least one medication classified as potentially inappropriate by the 2012 Beers Criteria. Additionally, 27% and 43% of the participants used at least one medication classified as “Avoid” and “Use Conditionally” by the 2012 Beers Criteria, respectively. Analyzing the 2006–2010 Medical Expenditure Panel Survey (MEPS), Davidoff and her colleagues documented that almost 43% of older adults with Rx had at least one medication fill that met the board definition of 2012 Beers Criteria [47]. Therefore, the number of PIMs used among our sample of African American older adults is higher than national averages reported by Davidoff and her colleagues.

Our study suggests that having multiple comorbidities and multiple providers (physicians and pharmacists) may be additional independent risk factors for polypharmacy. More than 28% of our sample of underserved, older African Americans used multiple pharmacies. The multivariate analysis of our data shows that when controlling for PIM, having multiple providers remains a significant correlate of polypharmacy. Switching between providers and pharmacies may exacerbate communication among patients, pharmacists, and physicians, increasing the risk of PIM and polypharmacy. Other researchers documented that filling prescriptions at multiple pharmacies is associated with lower medication adherence across multiple chronic medications and a greater likelihood of drug-drug interactions in concurrent pharmacy users [48]. Therefore, pharmacists serving older adults in underserved communities need to be aware that their patients may be using multiple pharmacies, especially those with multiple providers and comorbidities, as well as those who may get some of their prescriptions filled at the pharmacy of free clinics.

Another finding of this study that needs additional attention is that older African American women in our sample are more likely to use a higher number of medications than their male counterparts. Our data show that 65% and 81% of men and women are taking ≥ 5 medications, respectively. In addition, 35% of women participating in this study were taking at least 10 medications. Furthermore, African American women in our sample are more likely than their male counterparts to use medications categorized as “potentially inappropriate” by the Beers Criteria (31% versus 19%); to use medication with drug-drug interactions (56% versus 47%); to use multiple providers (73% versus 62%); and have more than three chronic conditions (63% versus 71%). However, using multivariate analysis, even after all the above-mentioned variables and other demographic characteristics were controlled for, women are still more likely to use a higher number of medications that men.

It is imperative to mention several limitations of this study. First, this study used a cross-sectional study design, which allowed us to collect data at a single point in time. Second, African Americans who participated in this study were selected from local churches that may be different than non-church attenders and this may introduce a selection bias. Third, the assumption is made that participants brought in all of the medication vials that they used within two weeks prior to the interviews. Lack of access to participants' medical or pharmacy records limits our ability to validate this assumption. In addition, participants were asked to bring medications used within the last two weeks; these may exclude medications dosed monthly or old medications patients find when they have a cold, and so on. However, several attempts were made prior to the face-to-face interviews to ensure that participants would follow the instructions and bring all their medications with them. Finally, our sample was generated from an underserved, urban African American community; the results of this study may not be generalizable to all African American older adults.

## 5. Conclusion

This study shows a high rate of polypharmacy and PIM use among our sample of underserved older African American adults residing in South Los Angeles. We documented a strong association between polypharmacy and PIM use, comorbidities, and having multiple providers. Exacerbation of these issues may be attributed to poor coordination and management of medications among providers and pharmacists. Therefore, there is a need to develop more innovative and effective strategies to reduce inappropriate polypharmacy [42]. Home-based, community-patient-centered, multidisciplinary approaches that connect local, community-based pharmacists with patients and their providers may be a promising approach. While a few researchers have suggested a multidisciplinary approach that coordinates medication use of older adults by providing both physicians and pharmacists with the ability to view online the prescription histories of patients [49], Lancaster and colleagues suggest that home-based, multidisciplinary assessments and reconciliation activities on an ongoing basis are needed to prevent medication related complications [50]. A Swedish study among older adults indicated that when reconciliation activities are mandated by law, the number of prescribed drugs and the extent of inappropriate drug therapy decreased significantly [51]. Regardless, patient and provider barriers to polypharmacy are numerous, and resources must be made available to facilitate deliberate yet judicious deprescribing among this segment of our population that needs it the most.

## Figures and Tables

**Table 1:** Characteristics of study sample by high versus low medication use (*N* = 400).

Characteristic of sample	*N* (%)	Groups of medication users		
		0–4 *N* (%) 100 (25)	≥5 *N* (%) 300 (75)	*p* Value
*Gender*				
Female	259 (65)	50 (19)	209 (81)	0.001
Male	141 (35)	50 (36)	91 (65)	
*Age*				
65–69	135 (34)	47 (35)	88 (65)	0.002
70–79	180 (45)	31 (17)	149 (83)	
≥80	85 (21)	22 (26)	63 (74)	
*Education*				
No high school diploma	99 (25)	19 (19)	80 (81)	0.078
High school diploma	301 (75)	81 (27)	220 (73)	
*Marital status*				
Married/living with partner	78 (20)	17 (22)	61 (78)	0.283
Not married	322 (80)	83 (26)	239 (74)	
*Copayment for RX*				
No	83 (21)	27 (33)	56 (67)	0.075
Yes	317 (79)	73 (23)	244 (77)	
*Number of providers*				
1-2	247 (62)	92 (37)	155 (63)	0.001
≥3	153 (38)	8 (5)	145 (95)	
*Number of pharmacies*				
1	287 (72)	82 (29)	205 (71)	0.009
≥2	113 (28)	18 (16)	95 (84)	
*Number of Comorbidity*				
≤3	128 (32)	60 (47)	68 (53)	0.001
4–7	195 (49)	34 (17)	161 (83)	
≥8	77 (19)	6 (8)	71 (92)	
*Smoking status*				
Current	81 (20)	14 (17)	67 (83)	0.001
Former	64 (16)	28 (44)	36 (56)	
Never	255 (64)	58 (23)	197 (77)	
*Alcohol use (drinks per day)*				
None	291 (73)	70 (24)	221 (76)	0.404
1-2	71 (18)	22 (31)	49 (69)	
≥3	38 (9)	8 (21)	30 (79)	
*Number of PIM*				
None	122 (31)	54 (44)	68 (56)	0.001
One	143 (36)	31 (112)	112 (78)	
≥2	135 (34)	15 (11)	120 (89)	
*Level of pain*				
None-mild	114 (29)	46 (40)	68 (60)	0.001
Moderate	84 (21)	22 (26)	62 (74)	
Severe	202 (50)	32 (16)	170 (84)	

**Table 2:** Multivariate logistic analysis of correlates of use of high versus low medication use (*N* = 400).

Independent variables	OR (95% CI)	*p*
*Gender*		
Female	2.36 (1.26–4.42)	0.007
Male	1	
*Age*		
65–69	0.69 (0.30–1.57)	0.067
70–79	1.53 (0.69–3.41)	0.374
≥80	1	0.297
*Education*		
No high school diploma	1.71 (0.82–3.56)	0.151
High school diploma	1	
*Marital status*		
Not married	2.21 (0.99–4.857)	0.501
Married/living with partner	1	
*Copayment for RX*		
No	1.14 (0.55–2.34)	0.725
Yes	1	
*Number of providers*		
1-2	1	0.001
≥3	6.67 (2.82–15.71)	
*Number of pharmacies*		
1	1	0.620
≥2	1.52 (0.73–3.17)	
*Number of comorbidity*		
≤3	1	0.001
4–7	3.20 (1.72–5.94)	0.001
≥8	6.11 (2.02–18.50)	0.064
*Smoking status*		
Never	1	0.852
Current	1.08 (0.49–2.38)	0.028
Former	0.38 (0.16–0.90)	0.338
*Alcohol use (drinks per day)*		
None	1	0.572
1-2	0.80 (0.37–1.74)	0.227
≥3	1.98 (0.65–6.01)	0.000
*Number of PIM*		
None	1	0.015
One	2.24 (1.17–4.30)	0.001
≥2	4.60 (2.10–10.04)	0.221
*Level of pain*		
None-mild	1	0.892
Moderate	0.95 (0.44–2.05)	0.136
Severe	1.70 (0.85–3.41)	

−2 log likelihood	303.6	
Nagelkerke *R*-square	0.454	
Percentage of correctly predicted outcome	82%	
